# A Comparison of Algorithms to Achieve the Maximum Entropy in the Theory of Evidence

**DOI:** 10.3390/e28020247

**Published:** 2026-02-21

**Authors:** Joaquín Abellán, Aina López-Gay, Maria Isabel A. Benítez, Francisco Javier G. Castellano

**Affiliations:** Department of Computer Science and Artificial Intelligence, University of Granada, 18071 Granada, Spain; ainalopez@correo.ugr.es (A.L.-G.); miab@correo.ugr.es (M.I.A.B.); fjgc@decsai.ugr.es (F.J.G.C.)

**Keywords:** evidence theory, reachable probability intervals, uncertainty measures, maximum of entropy

## Abstract

Within the framework of evidence theory, maximum entropy is regarded as a measure of total uncertainty that satisfies a comprehensive set of mathematical properties and behavioral requirements. However, its practical applicability is severely questioned due to the high computational complexity of its calculation, which involves the manipulation of the power set of the frame of discernment. In the literature, attempts have been made to reduce this complexity by restricting the computation to singleton elements, leading to a formulation based on reachable probability intervals. Although this approach relies on a less specific representation of evidential information, it has been shown to provide an equivalent maximum entropy value under certain conditions. In this paper, we present an experimental comparative study of two algorithms for calculating maximum entropy in evidence theory: the classical algorithm, which operates directly on belief functions, and an alternative algorithm based on reachable probability intervals. Through numerical experiments, we demonstrate that the differences between these approaches are less pronounced than previously suggested in the literature. Depending on the type of information representations to which it is applied, the original algorithm based on belief functions can be more efficient than the one using the reachable probability interval approach. This is an interesting result, and a reason for choosing one algorithm over the other depending on the situation.

## 1. Introduction

The representation and quantification of uncertainty are central issues in information theory, artificial intelligence, and decision-making under incomplete or imprecise knowledge. Classical probability theory (PT) provides a rigorous and widely accepted framework for uncertainty modeling; however, it requires precise probability assignments that are often unavailable in real-world applications. This limitation has motivated the development of more general models of uncertainty, commonly referred to as theories based on imprecise probabilities [1].

Among these frameworks, evidence theory (ET), also known as Dempster–Shafer theory [2,3], has been extensively employed to manage uncertainty-based information in practical applications such as medical diagnosis [4], statistical classification [5], target identification [6], and face recognition [7]. Evidence theory extends PT by introducing the concept of a *basic probability assignment* (BPA), which generalizes the notion of a probability distribution. Each BPA induces an associated belief function and a plausibility function, where the belief (respectively, plausibility) of a set represents the minimum (respectively, maximum) degree of support that the available evidence provides for that set.

Quantifying the uncertainty represented by a BPA is a fundamental problem in evidence theory. To this end, numerous uncertainty measures have been proposed, most of which are inspired by Shannon entropy [8], the standard measure of uncertainty in PT. However, extending Shannon entropy to evidence theory is non-trivial, as ET accounts for additional types of uncertainty not present in classical probability models.

As pointed out by Yager [9], two different types of uncertainty arise in evidence theory: *conflict*, which occurs when information supports disjoint sets, and *non-specificity*, which arises when information is assigned to sets with cardinality greater than one. Consequently, any uncertainty measure in ET must be able to jointly capture both conflict and non-specificity in a coherent manner.

Klir and Wierman [10] analyzed the mathematical properties that uncertainty measures in evidence theory should satisfy; this study was later extended by Abellán and Masegosa [11], who also introduced behavioral requirements for such measures. Among all proposals to date, the maximum entropy defined on the closed and convex set of probability distributions (credal set) compatible with a BPA [10] is the only uncertainty measure in evidence theory that satisfies all crucial mathematical properties and behavioral requirements simultaneously.

The Maximum Entropy Principle [12] is applicable in various domains, being principally related to information theory and applications [13,14,15]. It states that, in the absence of complete information, one should prioritize the most unbiased distribution by maximizing uncertainty, thereby ensuring that no groundless assumptions are introduced into the model. In the fields of evidence theory and uncertainty quantification [16,17,18,19,20], this principle and its associated uncertainty measures are essential for constructing belief distributions that reflect incomplete or ambiguous information.

Despite its strong axiomatic foundations, the practical computation of maximum entropy in evidence theory remains challenging. The algorithm proposed in [21] (also presented in [19]) involves solving constrained optimization problems whose complexity grows exponentially with the size of the frame of discernment. Consequently, numerous alternative uncertainty measures with lower computational complexity have been proposed in recent years. However, these alternatives often fail to satisfy all the required axiomatic properties and behavioral conditions [22,23,24], contributing to a lack of consensus regarding uncertainty measures in ET. In summary, while maximum entropy exhibits optimal axiomatic behavior, it entails a high computational cost; conversely, alternative measures are computationally efficient but theoretically weaker.

On the other hand, maximum entropy has demonstrated excellent performance in practical applications, particularly in data mining and related fields. For specific classes of belief functions, its computation becomes straightforward and can be executed efficiently, as illustrated in [15,25,26].

An approximation of the maximum entropy of the credal set associated with a BPA was proposed in [27]. This approach computes the maximum entropy over the credal set consistent with belief intervals for singleton elements, where the lower and upper bounds correspond to belief and plausibility values, respectively. Although this measure satisfies all crucial mathematical properties and behavioral requirements, utilizing belief intervals for singletons instead of the full BPA may lead to information loss. Indeed, the credal set associated with a BPA is always contained within the credal set that is compatible with the corresponding belief intervals [27], resulting in an uncertainty measure that may overestimate the level of uncertainty represented by the original BPA. However, this method offers the advantage of bypassing the enumeration of all subsets of the frame of discernment. A key question remains regarding whether this computational advantage persists across all possible scenarios.

The aim of this paper is to provide a comparative study of two algorithms for computing maximum entropy in the theory of evidence: the Meyerowitz et al. algorithm [19,21], which operates directly on belief functions; and the maximum entropy algorithm based on reachable probability intervals [18,27], derived from evidential constraints. The comparison addresses both theoretical aspects and numerical behavior, highlighting the advantages and limitations of each approach under various uncertainty scenarios.

The remainder of this paper is organized as follows. Section 2 introduces the basic concepts of evidence theory, reviews the main uncertainty measures proposed in this framework, and describes the algorithms for computing maximum entropy from a BPA and from belief intervals for singletons. Section 3 presents the comparative study via numerical examples and experiments in which millions of different belief functions are randomly generated. Finally, concluding remarks and directions for future work are provided in Section 4.

## 2. Background

Let $X=\{x_{1},\ldots ,x_{n}\}$ be a finite set of possible alternatives, also known as the *frame of discernment*. Let ℘(X) denote the power set of *X*.

### 2.1. Theory of Evidence

Evidence theory (ET), also known as Dempster–Shafer theory [2,3], is based on the concept of a *basic probability assignment* (BPA). A BPA is a mapping m:℘(X)→[0,1] satisfying m(∅)=0 and $\sum_{A\subseteq X}m\left(A\right)=1$.

If A⊆X satisfies m(A)>0, then *A* is said to be a *focal element* of *m*.

A given BPA *m* on *X* has an associated *belief function* $Bel_{m}$ and a *plausibility function* $Pl_{m}$. These functions are defined as follows:

(1) $$Bel_{m}\left(A\right)=\sum_{B\subseteq A}m\left(B\right),\;Pl_{m}\left(A\right)=\sum_{B\cap A\neq \emptyset }m\left(B\right),\;\forall A\subseteq X.\;$$

It should be noted that for each A⊆X, $Bel_{m}\left(A\right)\leq Pl_{m}\left(A\right)$. The interval $\left[Bel_{m}\left(A\right),Pl_{m}\left(A\right)\right]$ is referred to as the *belief interval* of *A*. Furthermore,

(2) $$Pl_{m}\left(A\right)=1-Bel_{m}\left(\bar{A}\right),\;\forall A\subseteq X,\;$$

where $\bar{A}$ denotes the complement of *A*. Consequently, $Bel_{m}$ and $Pl_{m}$ are considered dual or conjugate functions. Either function is sufficient to represent uncertainty-based information in ET; for this purpose, $Bel_{m}$ is more commonly utilized.

For a given BPA *m* on *X*, the set of compatible probability distributions (which corresponds to a closed and convex set, also known as a *credal set*) is defined as follows:

(3) $$P_{m}=\left\{p\in P\left(X\right)|Bel_{m}\left(A\right)\leq p\left(A\right)\;\forall A\subseteq X\right\},\;$$

where P(X) is the set of all probability distributions on *X*.

### 2.2. Uncertainty Measures in Evidence Theory

Shannon entropy [8] is the standard uncertainty measure in probability theory. Given a probability distribution *p* defined on a finite set $X=\{x_{1},\ldots ,x_{n}\}$, Shannon entropy is defined as follows:

(4) $$S\left(p\right)=-\sum_{i=1}^{n}p\left(\left\{x_{i}\right\}\right)\log_{2}\left(p\left(\left\{x_{i}\right\}\right)\right).$$

The type of uncertainty quantified by *S* is usually referred to as *conflict*, which is the only form of uncertainty present in classical probability theory. Shannon entropy satisfies a well-known set of desirable axiomatic properties [8,10].

In possibility theory, uncertainty is commonly quantified by the Hartley measure [28], defined as follows:

(5) $$H\left(A\right)=\log_{2}\left|A\right|,\;\forall A\in ℘\left(X\right).\;$$

This measure captures *non-specificity*, the sole type of uncertainty considered in possibility theory.

As noted by Yager [9], conflict and non-specificity coexist in ET. Conflict arises when information supports mutually exclusive subsets, whereas non-specificity appears when information is assigned to sets with cardinality greater than one. A generalization of the Hartley measure to evidence theory was introduced by Dubois and Prade [29]:

(6) $$GH\left(m\right)=\sum_{A\subseteq X}m\left(A\right)\log_{2}\left|A\right|.\;$$

GH attains its minimum value (zero) when *m* is a probability distribution, and its maximum value ($\log_{2}n$) when m(X)=1. It constitutes an appropriate measure of non-specificity in ET and can be naturally extended to more general uncertainty frameworks [18].

Numerous attempts have been made to generalize Shannon entropy to evidence theory; however, most proposals fail to satisfy the essential mathematical and behavioral requirements for this framework. A total uncertainty measure capable of jointly capturing conflict and non-specificity was proposed by Harmanec and Klir [19]. This measure, denoted by $S^{*}\left(P_{m}\right)$, is defined as the maximum Shannon entropy over the credal set $P_{m}$ associated with a BPA *m*:

(7) $$S^{*}\left(P_{m}\right)=\max_{p\in P_{m}}S\left(p\right).\;$$

To date, this is the only measure that satisfies all necessary mathematical properties and behavioral requirements for uncertainty measures in evidence theory [19,26].

Despite its strong foundations, computing $S^{*}\left(P_{m}\right)$ is computationally demanding. Algorithms proposed in the literature [19,21,30,31] involve solving nonlinear optimization problems with exponential complexity. Consequently, several alternative measures with lower computational costs have been proposed.

One well-known alternative is Deng entropy [22,32,33,34], defined as follows:

(8) $$E_{d}\left(m\right)=-\sum_{A\subseteq X}m\left(A\right)\log_{2}\left(\frac{m(A)}{2^{\left|A\right|}-1}\right).\;$$

In this formulation, the expression can be decomposed into two components: one capturing non-specificity and the other quantifying conflict. However, Deng entropy violates several essential mathematical properties and exhibits problematic behavior in various scenarios [23]. Similarly, Pan [35] introduced an uncertainty measure based on the plausibility transformation:

(9) $$H_{PQ}\left(m\right)=-\sum_{A\subseteq X}m\left(A\right)\log_{2}Pm\left(A\right)+GH\left(m\right),\;$$

where $Pm\left(A\right)=\sum_{x\in A}Pt\left(x\right)$, and Pt(x) is the *plausibility transformation* value. As shown in [27], $H_{PQ}$ also fails to satisfy all required mathematical properties.

Let us now consider the set of belief intervals for singleton elements associated with a BPA *m*:

(10) $$I_{m}=\left\{\left[Bel_{m}\left(\left\{x_{i}\right\}\right),Pl_{m}\left(\left\{x_{i}\right\}\right)\right]|i=1,\ldots ,n\right\}.$$

Zhao et al. [36] proposed a measure ($H_{inter}$) combining Deng entropy with these intervals. Nevertheless, it does not satisfy all crucial mathematical properties required in ET [27].

Let $P(I_{m})$ denote the credal set consistent with the belief intervals for singleton elements:

(11) $$P\left(I_{m}\right)=\left\{p\in P\left(X\right)|Bel_{m}\left(\left\{x_{i}\right\}\right)\leq p\left(\left\{x_{i}\right\}\right)\leq Pl_{m}\left(\left\{x_{i}\right\}\right),\;i=1,\ldots ,n\right\}.\;$$

In [27], a new uncertainty measure was proposed as the maximum entropy over $P(I_{m})$:

(12) $$S^{*}\left(P\left(I_{m}\right)\right)=\max_{p\in P(I_{m})}S\left(p\right).\;$$

This measure satisfies all essential requirements [27]. However, since $P_{m}\subseteq P\left(I_{m}\right)$, representing uncertainty through singleton belief intervals may result in information loss. Consequently, $S^{*}\left(P\left(I_{m}\right)\right)$ may indicate a higher level of uncertainty than the original BPA. Its primary advantage lies in the significant reduction of computational complexity, albeit at the cost of potential information loss.

### 2.3. Maximum Entropy from a Belief Function

The maximum entropy associated with a belief function is obtained by solving a constrained optimization problem over the credal set induced by a basic probability assignment. An exact procedure for this task was proposed by Meyerowitz et al. [21] and subsequently by Harmanec and Klir [19]. The algorithm iteratively constructs the probability distribution that maximizes Shannon entropy while satisfying the evidential constraints. Algorithm 1 calculates the maximum entropy of a BPA *m* with associated Bel() function. The procedure is described as follows:
**Algorithm 1** Algorithm to attain the maximum entropy from a Belief function X← current frame of discernment Bel← associated belief function **while** X≠∅ **and** 
Bel(X)≠0 
**do**      Select a non-empty subset A⊆X maximizing $\frac{Bel(A)}{\left|A\right|}$      If multiple subsets satisfy the condition, select the one with maximum cardinality      **for** x∈A **do**           Assign probability $\frac{Bel(A)}{\left|A\right|}$ to *x*      **end for**      **for** B⊆X∖A **do**           $Bel^{\prime }\left(B\right)\leftarrow Bel\left(B\cup A\right)-Bel\left(A\right)$      **end for**      X←X∖A      $Bel\leftarrow Bel^{\prime }$ **end while** **if** 
X≠∅ 
**then**      **for** x∈X **do**           Assign probability 0 to *x*      **end for** **end if**

The resulting probability distribution maximizes Shannon entropy under the constraints induced by the original belief function. Although exact, this algorithm requires evaluating all subsets of the frame of discernment at each iteration, resulting in exponential computational complexity.

### 2.4. Maximum Entropy from Reachable Probability Intervals

An alternative approach to computing maximum entropy is based on the reachable probability intervals [1] derived from the belief and plausibility values of singleton elements. Let

$$I={\left\{\left[l_{i},u_{i}\right]\right\}}_{i=1}^{n}\;$$

denote the set of such intervals, where $l_{i}=Bel_{m}\left(\left\{x_{i}\right\}\right)$ and $u_{i}=Pl_{m}\left(\left\{x_{i}\right\}\right)$.

This algorithm builds upon the work in [18] to obtain the maximum entropy on the credal set associated with a reachable set of probability intervals. We introduce the following notation:

- $Min(p,In_{x})$: the minimum value of the probability distribution *p* among the components whose indices belong to the set $In_{x}$.
- $Sig(p,In_{x})$: the second smallest value of the probability distribution *p* among the components in $In_{x}$. If no such value exists, $Sig(p,In_{x})=-1$.
- $N_{min}\left(p,In_{x}\right)$: the number of indices in $In_{x}$ that attain the minimum value of the probability distribution *p*.
- Min(a,b,c): the minimum value among the real numbers {a,b,c}.

The following procedure (Algorithm 2) yields the probability distribution $\hat{p}$ that attains the maximum entropy on $P(I_{m})$:
**Algorithm 2** Algorithm to attain the maximum entropy from reachable probability intervals $index\_set\leftarrow \left\{1,2\ldots ,n\right\}$ **for** i=1 to *n* **do**      $\hat{p}\left(\left\{x_{i}\right\}\right)\leftarrow Bel_{m}\left(\left\{x_{i}\right\}\right)$ **end for** $mass\leftarrow 1-\sum_{i=1}^{n}Bel_{m}\left(\left\{x_{i}\right\}\right)$ **while** 
mass>0 **do**      **for** i∈index_set **do**           **if** $\hat{p}\left(\left\{x_{i}\right\}\right)=Pl_{m}\left(\left\{x_{i}\right\}\right)$ **then**                $index\_set\leftarrow index\_set\setminus \left\{i\right\}$           **end if**      **end for**      $min\leftarrow Min(\hat{p},index\_set)$      $second\leftarrow Sig(\hat{p},index\_set)$      $m\leftarrow N_{min}\left(\hat{p},index\_set\right)$      **for** i∈index_set **do**           **if** $\hat{p}\left(\left\{x_{i}\right\}\right)=Min\left(\hat{p},index\_set\right)$ **then**             **if** second=−1 **then**                  $\hat{p}\left(\left\{x_{i}\right\}\right)\leftarrow \hat{p}\left(\left\{x_{i}\right\}\right)+Min\left(Pl\left(\left\{x_{i}\right\}\right)-\hat{p}\left(\left\{x_{i}\right\}\right),\frac{mass}{m},1\right)$                  $mass\leftarrow mass-Min\left(Pl\left(\left\{x_{i}\right\}\right)-\hat{p}\left(\left\{x_{i}\right\}\right),\frac{mass}{m},1\right)$             **else**                  $\hat{p}\left(\left\{x_{i}\right\}\right)\leftarrow \hat{p}\left(\left\{x_{i}\right\}\right)+$$Min\left(Pl\left(\left\{x_{i}\right\}\right)-\hat{p}\left(\left\{x_{i}\right\}\right),second-\hat{p}\left(\left\{x_{i}\right\}\right),\frac{mass}{m}\right)$                  mass←mass− $Min\left(Pl\left(\left\{x_{i}\right\}\right)-\hat{p}\left(\left\{x_{i}\right\}\right),second-\hat{p}\left(\left\{x_{i}\right\}\right),\frac{mass}{m}\right)$             **end if**           **end if**      **end for** **end while**

Unlike the belief-function-based algorithm, this approach exhibits polynomial computational complexity relative to the number of singleton elements.

### 2.5. Discussion

The two algorithms analyzed in this work address the same conceptual objective—computing maximum entropy within the framework of evidence theory—but rely on fundamentally different representations of uncertainty. Meyerowitz et al.’s algorithm operates directly on belief functions and computes the exact maximum entropy associated with the credal set induced by a BPA. In contrast, the interval-based algorithm computes maximum entropy over a larger credal set defined solely by the belief and plausibility values of singleton elements.

From a theoretical perspective, the Meyerowitz et al. algorithm preserves the full informational content of the belief function, providing an exact characterization of total uncertainty. However, this precision entails high computational complexity that grows exponentially with the size of the frame of discernment. Consequently, its applicability is primarily limited to problems of moderate dimensionality.

The interval-based algorithm significantly reduces computational overhead by restricting the optimization problem to singleton constraints. Although this approach may result in information loss—since the credal set induced by singleton intervals contains only the one associated with the original belief function—the experimental results demonstrate that the resulting maximum entropy values are often remarkably close or even identical to those obtained by the exact algorithm. This suggests that, in many practical scenarios, the loss of specificity introduced by interval representations has a negligible impact on the total uncertainty measure.

Contrary to claims in previous studies, our analysis indicates that the discrepancy between the two approaches is not as pronounced as might be expected. The interval-based method provides a reliable approximation of maximum entropy while offering substantial computational advantages. These findings underscore the importance of balancing representational accuracy with computational feasibility when selecting algorithms for evidence-based models.

## 3. Comparison of Algorithms

Our principal aim in this work is to compare the performance of the maximum entropy calculation algorithms.

We will introduce several examples and apply the respective algorithms to them in order to compare them. The objective of this comparison is to see how both algorithms work in a specific situation when both are applied within the TE. To do this, we will first study them by applying Meyerowitz et al.’s algorithm, specific to the theory of evidence, with the algorithm adapted to reachable intervals, taking only the singletons. We remark that the calculus of the values of Bel(), Pl(), $l_{i}$ and $u_{i}$ is based on the expressions in the Section 2.

The first two examples are free of conflict, while the following two have conflict, allowing us to see how this situation could affect the effectiveness of the algorithms. To simplify, *Algorithm-1* is the algorithm of Meyerowitz et al., and *Algorithm-2* is the algorithm based on the reachable probability intervals of the singletons.

**Example** **1.** *Let us start with the set X={a,b,c,d,e}, whose mass function m is given by*

$$\begin{array}{cc} m(\{a,b\}) & =0.4\\ m(\{c,d\}) & =0.35\\ m(\{e\}) & =0.25 \end{array}$$

*We see that subsets {a,b},{c,d}, and {e} of X are mutually disjointed, so there is no conflict. With this, we will proceed to apply the algorithms presented above.*

*1.*  ***Algorithm-1.***
*We first construct Table 1 with the values of the belief function for each subset A⊂X and the value of $\frac{Bel(A)}{\left|A\right|}$.*
  - *First iteration.*
*We observe that the maximum value of $\frac{Bel(A)}{\left|A\right|}$ is for A={e}. With this, we have $p_{e}=0.25$, and we can make modifications to the belief functions so that we obtain*
Bel({a,b})←Bel({a,b,e})−Bel({e})=0.65−0.25=0.4,
Bel({a,b,c,d})←Bel({a,b,c,d,e})−Bel({e})=1−0.25=0.75.
*Now, we take X←{a,b,c,d,e}−{e}={a,b,c,d},X≠∅ and Bel(X)≠∅, performing a new iteration.*
  - *Second iteration.*
*We start from X={a,b,c,d}, so we have the values seen in Table 2:*
    **Table 2:** Values of Bel(A) and $\frac{Bel(A)}{\left|A\right|}$ in the second iteration.

*A*	*Bel(A)*	$\frac{\mathrm{Bel}(A)}{\left|A\right|}$
{a,b}	0.40	0.20
{c,d}	0.35	0.175
{a,b,c}	0.40	0.1$\bar{3}$
{a,b,d}	0.40	0.1$\bar{3}$
{a,c,d}	0.35	0.11$\bar{6}$
{b,c,d}	0.35	0.11$\bar{6}$
{a,b,c,d}	0.75	0.1875

*In this case, the maximum of $\frac{Bel(A)}{\left|A\right|}$ is reached at $\frac{Bel(\{a,b\})}{\left|\{a,b\}\right|}$, so we assign $p_{a}=p_{b}=0.20$. Moving to the next step of the algorithm, X←{a,b,c,d}−{a,b}={c,d}≠∅, and*
Bel({c,d})←Bel({a,b,c,d})−Bel({a,b})=0.75−0.40=0.35≠∅,
    *so a new iteration begins.*
  - *Third iteration.*
*Given that X={c,d}, the only possible non-zero value is that shown in Table 3:*
    **Table 3:** Values of Bel(A) and $\frac{Bel(A)}{\left|A\right|}$ in the third iteration.

*A*	*Bel(A)*	$\frac{\mathrm{Bel}(A)}{\left|A\right|}$
{c,d}	0.35	0.175

*Thus, the maximum of $\frac{Bel(A)}{\left|A\right|}$ is A={c,d}. Thus, $p_{c}=p_{d}=0.175$ and we obtain the new X={c,d}−{c,d}=∅, with Bel(∅)=0, so we can proceed to calculate the maximum entropy.*
$$S^{*}\left(Bel\right)=-\sum_{i\in \{a,b,c,d,e\}}p_{i}\log_{2}\left(p_{i}\right)=-(2\cdot 0.2\cdot \log_{2}\left(0.2\right)+$$
$$+2\cdot 0.175\cdot \log_{2}\left(0.175\right)+0.25\cdot \log_{2}\left(0.25\right))=\begin{array}{c} 2.308872 \end{array}$$
*2.*  ***Algorithm-2***
*To apply this algorithm, we need to calculate the probability intervals of the singletons. Calculating the belief and plausibility functions associated with each element of {a,b,c,d,e}, we obtain the values shown in Table 4:*
*Therefore, we start with the following set of probability intervals*
L={[0,0.4],[0,0.4],[0,0.35],[0,0.35],[0.25,0.25]}
  *where:*
l=[0,0,0,0,0.25],u=[0.40,0.40,0.35,0.35,0.25]
  *and $\hat{p}$ is the probability vector for which we will calculate the maximum entropy.*
  - *First iteration.*
*To begin, we initialize with S={1,2,3,4,5}, where *1* corresponds to singleton a, *2* to singleton b, etc. We construct the vector $\hat{p}$ with the lower bound values $l_{i}$ for each i∈{1,2,3,4,5}:*
$$\hat{p}=\left(0,0,0,0,0.25\right)$$
    *and we see that $Sum(\hat{p})=0.25<1$. We check if $l_{i}=u_{i}$ for some i=1,...,5, and we see that it holds for i=5, so we remove it from S, obtaining S={1,2,3,4}. We calculate the following values:*
    * *$s=Sum(l_{i})=0.25$,*
    * *r=Min(l,S)=1,*
    * *m=Nmin(l,S)=4,*
    * *Sig(l,S)=−1,*
    *hence, using the algorithm, we carry out the assignment*
$$l_{i}⟵l_{i}+\min\left(u_{i}-l_{i},\frac{1-s}{m},1\right)=l_{i}+0.1875,$$
    *updating ${\hat{p}}_{i}$ for each i=1,...,5, so we have*
*$\hat{p}=\left[0.1875,0.1875,0.1875,0.1875,0.25\right]$.*
  - *Second iteration.*
*We start from*
$$\hat{p}=\left(0.1875,0.1875,0.1875,0.1875,0.25\right),$$
    *with $Sum(\hat{p})=1$, so the algorithm ends and we proceed to calculate the maximum entropy associated with the given distribution:*
$$S^{*}\left(Bel\right)=-\left(4\cdot 0.1875\right)\log_{2}\left(0.1875\right)+0.25\cdot \log_{2}\left(0.25\right))=\begin{array}{c} 2.311278 \end{array}$$

**Example** **2.** *We consider the set X={a,b,c,d,e,f} with a given mass function m defined by*

$$\begin{array}{cc} m(\{a\}) & =0.1\\ m(\{b,c\}) & =0.4\\ m(\{d,f\}) & =0.35\\ m(\{e\}) & =0.15 \end{array}$$

*1.*  ***Algorithm-1***
*We have the values for the belief function and $\frac{Bel(A)}{\left|A\right|}$ for each subset A⊆X, as shown in Table 5:*
  - *First iteration.*
*We observe that the maximum of $\frac{Bel(A)}{\left|A\right|}$ is reached for A={b,c}. Thus, for the elements of this set, we have $p_{b}=0.2$ and $p_{c}=0.20$. With this, we proceed to update the values of the belief function as follows:*
Bel({d,f})←Bel({b,c,d,f})−Bel({b,c})=0.50−0.40=0.10,
Bel({a,d,e,f})←Bel({a,b,c,d,e,f})−Bel({b,c})=1−0.40=0.60.
*We update X={a,d,e,f}≠∅, verifying that Bel(X)≠0, and apply the algorithm again.*
  - *Second iteration.*
*We start from X={a,d,e,f} with the values in Table 6:*
    **Table 6:** Values of Bel(A) and $\frac{Bel(A)}{\left|A\right|}$ in the second iteration.

*A*	*Bel(A)*	$\frac{\mathrm{Bel}(A)}{\left|A\right|}$
{a}	0.10	0.10
{e}	0.15	0.15
{a,d}	0.10	0.05
{a,e}	0.25	0.125
{a,f}	0.10	0.05
{d,e}	0.15	0.075
{d,f}	0.35	0.175
{e,f}	0.15	0.075
{a,d,e}	0.25	0.08$\bar{3}$
{a,d,f}	0.45	0.15
{a,e,f}	0.25	0.08$\bar{3}$
{d,e,f}	0.50	0.1$\bar{6}$
{a,d,e,f}	0.60	0.15

*In this case, the maximum of $\frac{Bel(A)}{\left|A\right|}$ is 0.175, a value that corresponds to the set {d,f}. Thus, $p_{d}=0.175=p_{f}$ and we can update the belief function:*
Bel({a,e})←Bel({a,d,e,f})−Bel({d,f})=0.6−0.35=0.25.
*So, our new set is X={a,e}≠∅ whose associated belief function is Bel(X)=0.25≠0; therefore, we begin another iteration.*
  - *Third iteration.*
*We have the values shown in Table 7:*

**Table 7:** Values of Bel(A) and $\frac{Bel(A)}{\left|A\right|}$ in the third iteration.

*A*	*Bel(A)*	$\frac{\mathrm{Bel}(A)}{\left|A\right|}$
{a}	0.10	0.10
{e}	0.15	0.15
{a,e}	0.25	0.125
*From these, we can see that $\frac{Bel(A)}{\left|A\right|}$ is maximized if A={e}, so we assign $p_{e}=0.15$. With this, we update the value of the function Bel:*
Bel({a})←Bel({a,e})−Bel({e})=0.25−0.15=0.1.
*We then have now that X={a}≠∅ with Bel(X)≠0, so the algorithm is applied again.*
  - *Fourth iteration.*
*Since we start from the set X={a}, we maximize $\frac{Bel(A)}{\left|A\right|}$ on this same set, so we assign $p_{a}=0.1$. By updating both the set X and the value of its belief function, we obtain X=∅ and Bel(X)=Bel(∅)=0, at which point the algorithm terminates, and we can proceed to obtain the value of the maximum entropy:*
$$S^{*}\left(Bel\right)=-(0.1\cdot \log_{2}\left(0.1\right)+0.15\cdot \log_{2}\left(0.15\right)+2\cdot 0.175\cdot \log_{2}\left(0.175\right)+$$
$$+2\cdot 0.2\cdot \log_{2}\left(0.2\right))=\begin{array}{c} 2.55151 \end{array}.$$
*2.*  ***Algorithm-2***
*First, we will transform the data given by the mass function into reachable intervals. The values of the belief function and the plausibility function associated with each element of {a,b,c,d,e,f} are as shown in Table 8:*
*Thus, we initialize the table to correctly apply the algorithm:*
l=[0.10,0,0,0,0.15,0],u=[0.10,0.40,0.40,0.35,0.15,0.35].
  - *First iteration.*
*We assign S={1,2,3,4,5,6}, and vector $\hat{p}$ is given by*
$$\hat{p}=\left[0.1,0,0,0,0.15,0\right],$$
    *which satisfies $Sum(\hat{p})=0.25<1$. We look for indices i such that $l_{i}=u_{i}$, with i=1,...,6. We have $l_{1}=u_{1}$ and $l_{5}=u_{5}$, so S becomes S={2,3,4,6}. Therefore,*
    * *$s=Sum(l_{i})=0.25$,*
    * *r=Min(l,S)=2,*
    * *m=Nmin(l,S)=4,*
    * *f=Sig(l,S)=−1.*
*Hence, we apply the assignment $l_{i}⟵l_{i}+\min\left(u_{i}-l_{i},\frac{1-s}{m},1\right)$, with m=Nmin(l,S)=4, s=0.25. Thus, $\frac{1-s}{m}=0.1875$, and our vector becomes*
*$\hat{p}=\left[0.10,0.1875,0.1875,0.1875,0.15,0.1875\right]$, before applying the algorithm again.*
  - *Second iteration.*
*We start with $\hat{p}=\left[0.10,0.1875,0.1875,0.1875,0.15,0.1875\right]$, where $Sum(\hat{p})=1$. The algorithm finishes, and we calculate the maximum associated entropy as follows:*
$$S^{*}\left(Bel\right)=-(0.10\cdot \log_{2}\left(0.10\right)+4\cdot 0.1875\cdot \log_{2}\left(0.1875\right)+$$
$$+0.15\cdot \log_{2}\left(0.15\right))=\begin{array}{c} 2.554145 \end{array}.$$

Unlike the two previous examples, we will now study two examples in which conflict appears.

**Example** **3.** *Given a set X={a,b,c,d}, we define the mass function m as*

$$\begin{array}{cc} m(\{a\}) & =0.15\\ m(\{b,c\}) & =0.40\\ m(\{c,d\}) & =0.30\\ m(\{a,d\}) & =0.15 \end{array}$$

*1.*  ***Algorithm-1***
*The values of the non-zero belief function associated with the A⊆X are shown in Table 9:*
  - *First iteration.*
*We identify that set A, which means the $\frac{Bel(A)}{\left|A\right|}$ maximal is {a,b,c,d}. Since the chosen set coincides with X, we assign probabilities to all its elements:*
$$p_{a}=p_{b}=p_{c}=p_{d}=0.25.$$
*Thus, for each B⊆X−A, we have Bel(B)=0, and the new set is set *∅*. Therefore, we can proceed to calculate the value of the maximum entropy associated with this distribution as follows:*
$$S^{*}\left(Bel\right)=-4\cdot 0.25\cdot \log_{2}\left(0.25\right)=\begin{array}{c} 2 \end{array}.$$
*2.*  ***Algorithm-2***
*For each element of {a,b,c,d,e}, we have its associated values, expressed in Table 10:*
*We can initialize the following:*
l=[0.15,0,0,0],u=[0.30,0.30,0.70,0.45].
  - *First iteration.*
*Let us assign S={1,2,3,4}, so for each i=1,2,3,4 we have the vector:*
$$\hat{p}=\left[0.15,0,0,0\right].$$
*It is true that Sum(l)=0.15<1, so we proceed to check if $l_{i}=u_{i}$ holds for some i. It does not hold for any i=1,2,3,4, so S={1,2,3,4}. We calculate the following:*
    * *$s=Sum(\hat{p})=0.15$,*
    * *r=Min(l,S)=2,*
    * *m=Nmin(l,S)=3,*
    * *f=Sig(l,S)=1, so we move to step 8.*
*We now assign $l_{i}+\min\left(u_{i}-l_{i},l_{f}-l_{r},\frac{1-s}{m}\right)⟶l_{i}$, where $\min\left(u_{i}-l_{i},l_{f}-l_{r},\frac{1-s}{m}\right)=0.15$*
  - *Second iteration.*
*The new vector $\hat{p}$ is given by:*
$$\hat{p}=\left[0.15,0.15,0.15,0.15\right],$$
    *verifying $Sum(\hat{p})=0.60<1$. Furthermore, no i∈{1,2,3,4} satisfies $l_{i}=u_{i}$, so S is not modified. Thus,*
    * *$s=Sum(\hat{p})=0.60$,*
    * *r=Min(l,S)=1,*
    * *m=Nmin(l,S)=4,*
    * *f=Sig(l,S)=−1.*
*Hence, we assign $l_{i}⟵l_{i}+\min\left(u_{i}-l_{i},\frac{1-s}{m},1\right)=l_{i}+\frac{1-0.60}{4}=l_{i}+0.10.$*
  - *Third iteration.*
*The vector $\hat{p}$ updated with the values from the previous iteration is as follows:*
$$\hat{p}=\left[0.25,0.25,0.25,0.25\right],$$
    *verifying $Sum(\hat{p})=1$, and thus terminating the algorithm. Thus, we have arrived at the fact that the probability vector of maximum entropy is $\hat{p}=\left[0.25,0.25,0.25,0.25\right]$, and we proceed to obtain the value of the maximum entropy:*
$$S^{*}\left(Bel\right)=-4\cdot 0.25\cdot \log_{2}\left(0.25\right)=\begin{array}{c} 2 \end{array}.$$

**Example** **4.** *We define the following mass function m on the set X={a,b,c,d,e}:*

$$\begin{array}{cc} m(\{a\}) & =0.10\\ m(\{b,c,d\}) & =0.35\\ m(\{d,e\}) & =0.20\\ m(\{a,c,e\}) & =0.25\\ m(\{b,e\}) & =0.10 \end{array}$$

*1.*  ***Algorithm-1***
*The values of Bel(A) for each A⊆X are shown in Table 11:*
  - *First iteration.*
*As in the previous example, we observe that the set that maximizes $\frac{Bel(A)}{\left|A\right|}$ is A={a,b,c,d,e}, and thus assign probabilities to all elements in the following form:*
$$p_{a}=p_{b}=p_{c}=p_{d}=p_{e}=0.20.$$
*We then proceed to take the set X=∅, with Bel(X)=0, so we move directly to calculating the maximum entropy:*
$$S^{*}\left(Bel\right)=-5\cdot 0.20\cdot \log_{2}\left(0.20\right)=\begin{array}{c} 2.3219 \end{array}.$$
*2.*  ***Algorithm-2***
*Table 12 shows the reachable interval associated with each element of {a,b,c,d,e}:*

*Therefore, we have:*

l=[0.10,0,0,0,0],u=[0.35,0.45,0.60,0.55,0.55].

- *First iteration.*
*We assign S={1,2,3,4,5}, $l_{i}⟶{\hat{p}}_{i}$, so that:*
$$\hat{p}=\left[0.10,0,0,0,0\right],$$
*$Sum(\hat{p})=0.10<1$. Furthermore, no i∈{1,2,3,4,5} satisfies $l_{i}=u_{i}$, so S remains the same. We now obtain the following values:*
  * *$s=Sum(\hat{p})=0.10$,*
  * *r=Min(l,S)=2,*
  * *m=Nmin(l,S)=4,*
  * *f=Sig(l,S)=1.*
*Since f=1, we perform the assignment presented in step 8, $l_{i}⟵l_{i}+\min\left(u_{i}-l_{i},l_{f}-l_{r},\frac{1-s}{m}\right)=l_{i}+min\left(0.45,0.10,0.225\right)=l_{i}+0.10$ and proceed to the next iteration.*
- *Second iteration*
*We now start from the vector:*
$$\hat{p}=\left[0.10,0.10,0.10,0.10,0.10\right],$$
  *such that $Sum(\hat{p})=0.50<1$. Again, no $l_{i}=u_{i}$ for i=1,2,...,5, then*
  * *$s=Sum(\hat{p})=0.50$;*
  * *r=Min(l,S)=1;*
  * *m=Nmin(l,S)=5;*
  * *f=Sig(l,S)=−1, so we go to step 7.*
*We assign $l_{i}⟵l_{i}+\min\left(u_{i}-l_{i},\frac{1-s}{m},1\right)=l_{i}+0.10$, and the algorithm is applied again.*
- *Third iteration.*
*Since we have $\hat{p}=\left[0.20,0.20,0.20,0.20,0.20\right]$, we can verify that $Sum(\hat{p})=1,$ thus terminating the algorithm. Therefore, the probabilities with maximum entropy for the given intervals are ${\hat{p}}_{a}={\hat{p}}_{b}={\hat{p}}_{c}={\hat{p}}_{d}={\hat{p}}_{e}=0.20$, and we calculate the associated maximum entropy:*
$$S^{*}\left(Bel\right)=-5\cdot 0.20\cdot \log_{2}\left(0.20\right)=\begin{array}{c} 2.3219 \end{array}.$$

Having seen these examples, we can highlight some differences between the algorithms. To do this, we will use Table 13:

From this first numerical comparison, we can make the following comments.

In the case of the examples where conflict appears, the number of iterations used by Algorithm-1 is lower than the number of iterations used by Algorithm-2. Furthermore, the maximum entropy coincides for both theories. Therefore, we might think that, for cases with conflict, Algorithm-1 is more efficient with respect to the number of iterations needed. However, in the case where there is no conflict, we can think that the opposite occurs.

In the following Subsection, we show what happens when we carry out an extensive experimentation with a very large number of different BPAs.

### Experimentation and Computational Analysis

To evaluate the computational efficiency of both algorithms, we conducted a series of experiments generating Basic Probability Assignments (BPAs) on frames of discernment with sizes n=5, n=6, and n=10. Both algorithms were implemented in the C++ programming language and executed on a system equipped with an Intel Core i5 1.8 GHz CPU and 8 GB of RAM.

To assess performance across different evidential structures, we randomly generated one million BPAs with conflict (C) and one million without conflict (NC). For conflicting cases (C), all possible subsets could serve as focal elements; values were generated in the range [0,100] and subsequently normalized. For the non-conflicting cases (NC), we considered combinations of disjoint sets. For n=5 and n=6, all possible disjoint combinations were explored, while for n=10, focal sets were restricted to cardinalities between 2 and 8 to maximize mass distribution. The results are summarized in Table 14.

The experimental results align with the theoretical expectations derived from the numerical examples. In scenarios involving conflict, where BPAs typically possess a larger number of focal elements, Algorithm-1 consistently outperforms Algorithm-2. This is noteworthy because a higher count of focal elements often presents a computational challenge. The relative underperformance of Algorithm-2 in these cases is attributed to the high overhead of calls to auxiliary functions. For n=6, Algorithm-1 shows an improvement of approximately 16.5% over Algorithm-2, a disparity that becomes even more pronounced at n=10, reaching a performance gain of 27.5%.

In contrast, in conflict-free scenarios, the performance hierarchy is partially reversed. Although the differences are negligible for n=5, Algorithm-2 shows an improvement of approximately 8% for n=6. In these cases, the number of focal sets is restricted by the disjointness constraint, which benefits the iterative structure of Algorithm-2.

For n=10, where the number of possible focal sets increases exponentially ($2^{10}-1=1023$), the behavioral patterns persist. In conflicting scenarios, Algorithm-1’s superiority suggests that its efficiency scales better with the number of elements in the universal set. In contrast, for non-conflict scenarios, both algorithms exhibit similar performance, with Algorithm-2 maintaining a slight advantage (below 3%). This convergence suggests that as *n* increases, the computational burden of calculating the Bel and Pl functions becomes the dominant factor in terms of overall complexity.

Theoretical analysis confirms these observations. Algorithm-1 solves a constrained maximization problem over the full credal set, leading to an exponential time and space complexity of $O(2^{n})$. Although Algorithm-2 employs a constructive strategy that appears polynomial ($O(n^{2})$), its execution time remains inherently linked to the number of focal sets |℘(X)|. When this number is large, the frequency of calls to auxiliary functions results in an overall exponential complexity comparable to that of Algorithm-1.

In conclusion, our experimental study suggests a pragmatic approach to algorithm selection: Algorithm-2 is preferable for belief structures where conflict is absent or minimal. However, for general applications where conflict is likely—a more common occurrence in real-world data—the classical Algorithm-1 remains the more robust and efficient choice.

## 4. Conclusions and Future Work

The computational cost associated with the algorithm of Meyerowitz et al. has traditionally been the primary drawback to using maximum entropy as a measure to quantify uncertainty and information within Evidence Theory (ET).

This paper critically analyzes the assertion that is frequently found in the recent literature [27]:

“The exact computation of maximum entropy in Evidence Theory, as performed by the algorithm of Meyerowitz et al. (Algorithm-1), is characterized by exponential computational complexity with respect to the size of the frame of discernment. In contrast, the interval-based formulation (Algorithm-2) reduces the problem to a polynomial-time optimization at the cost of a controlled loss of information. Since Algorithm-2 typically yields maximum entropy values close to those obtained with Algorithm-1, it constitutes a preferable alternative in practical applications, particularly for large frames of discernment.”

Our analysis reveals that when evaluating these algorithms, it is essential to distinguish between scenarios where conflict is absent and those where it is present. Theoretically, Algorithm-1 is expected to perform worse in the presence of conflict, as conflict typically increases the number of focal elements. Under such conditions, it has been generally assumed that Algorithm-2 would be more efficient. However, our experimental results demonstrate that this expected behavior does not consistently occur. This discrepancy can be attributed to the extensive number of function calls required by Algorithm-2, which effectively offsets its theoretical computational advantages.

As future work, we intend to investigate alternative procedures for computing maximum entropy in ET that offer superior computational efficiency, even if this entails obtaining approximate rather than exact values, as is currently the case with Algorithm-2.

Overall, the findings of this study challenge the common assumption that transforming a belief function into an equivalent representation based on reachable probability intervals necessarily facilitates the computation of maximum entropy.

## Figures and Tables

**Table 1:** Values of Bel(A) and $\frac{Bel(A)}{\left|A\right|}$.

*A*	*Bel(A)*	$\frac{\mathrm{Bel}(A)}{\left|A\right|}$
{a}	0	0
{b}	0	0
{c}	0	0
{d}	0	0
{e}	0.25	0.25
{a,b}	0.40	0.20
{a,c}	0	0
{a,d}	0	0
{a,e}	0.25	0.125
{b,c}	0	0
{b,d}	0	0
{b,e}	0.25	0.125
{c,d}	0.35	0.175
{c,e}	0.25	0.125
{d,e}	0.25	0.125
{a,b,c}	0.40	0.1$\bar{3}$
{a,b,d}	0.40	0.1$\bar{3}$
{a,b,e}	0.65	0.21$\bar{6}$
{a,c,d}	0.35	0.11$\bar{6}$
{a,c,e}	0.25	0.08$\bar{3}$
{a,d,e}	0.25	0.08$\bar{3}$
{b,c,d}	0.35	0.11$\bar{6}$
{b,c,e}	0.25	0.08$\bar{3}$
{b,d,e}	0.25	0.08$\bar{3}$
{c,d,e}	0.60	0.2
{a,b,c,d}	0.75	0.1875
{a,b,c,e}	0.65	0.1625
{a,b,d,e}	0.65	0.1625
{a,c,d,e}	0.60	0.15
{b,c,d,e}	0.6	0.15
{a,b,c,d,e}	1	0.20

**Table 4:** Belief intervals.

$x_{i}$	$\mathrm{Bel}{(x}_{i})$	$\mathrm{Pl}{(x}_{i})$	$\left[\mathrm{Bel}{(x}_{i}),\mathrm{Pl}{(x}_{i})\right]$
{a}	0	0.40	[0, 0.40]
{b}	0	0.40	[0, 0.40]
{c}	0	0.35	[0, 0.35]
{d}	0	0.35	[0, 0.35]
{e}	0.25	0.25	[0.25, 0.25]

**Table 5:** Values of Bel(A) and $\frac{Bel(A)}{\left|A\right|}$.

*A*	*Bel(A)*	$\frac{\mathrm{Bel}(A)}{\left|A\right|}$
{a}	0.10	0.10
{e}	0.15	0.15
{a,b}	0.10	0.05
{a,c}	0.10	0.05
{a,d}	0.10	0.05
{a,e}	0.25	0.125
{a,f}	0.10	0.05
{b,c}	0.40	0.20
{b,e}	0.15	0.075
{c,e}	0.15	0.075
{d,e}	0.15	0.075
{d,f}	0.35	0.175
{e,f}	0.15	0.075
{a,b,c}	0.50	0.1$\bar{6}$
{a,b,e}	0.25	0.08$\bar{3}$
{a,c,e}	0.25	0.08$\bar{3}$
{a,d,f}	0.45	0.15
{a,e,f}	0.25	0.08$\bar{3}$
{b,c,d}	0.40	0.1$\bar{3}$
{b,c,e}	0.55	0.18$\bar{3}$
{b,c,f}	0.40	0.1$\bar{3}$
{b,d,f}	0.35	0.11$\bar{6}$
{b,e,f}	0.15	0.05
{c,d,f}	0.35	0.11$\bar{6}$
{c,e,f}	0.15	0.15
{d,e,f}	0.50	0.1$\bar{6}$
{a,b,c,d}	0.50	0.125
{a,b,c,e}	0.65	0.1625
{a,b,c,f}	0.50	0.125
{a,b,d,f}	0.45	0.1125
{a,b,e,f}	0.25	0.0625
{a,c,d,f}	0.45	0.1125
{a,c,e,f}	0.25	0.0625
{a,d,e,f}	0.60	0.15
{b,c,d,e}	0.55	0.1375
{b,c,d,f}	0.75	0.1875
{b,c,e,f}	0.55	0.1375
{b,d,e,f}	0.50	0.125
{c,d,e,f}	0.50	0.125
{a,b,c,d,e}	0.65	0.13
{a,b,c,d,f}	0.85	0.17
{a,b,c,e,f}	0.65	0.13
{a,b,d,e,f}	0.60	0.12
{a,c,d,e,f}	0.60	0.12
{b,c,d,e,f}	0.9	0.18
{a,b,c,d,e,f}	1	0.1$\bar{6}$

**Table 8:** Belief intervals.

$x_{i}$	$\mathrm{Bel}{(x}_{i})$	$\mathrm{Pl}{(x}_{i})$	$\left[\mathrm{Bel}{(x}_{i}),\mathrm{Pl}{(x}_{i})\right]$
{a}	0.1	0.10	[0.10, 0.10]
{b}	0	0.40	[0, 0.40]
{c}	0	0.40	[0, 0.40]
{d}	0	0.35	[0, 0.35]
{e}	0.15	0.15	[0.15, 0.15]
{f}	0	0.35	[0, 0.35]

**Table 9:** Values of Bel(A) and $\frac{Bel(A)}{\left|A\right|}$.

*A*	*Bel(A)*	$\frac{\mathrm{Bel}(A)}{\left|A\right|}$
{a}	0.15	0.15
{b,c}	0.40	0.20
{c,d}	0.30	0.15
{a,d}	0.30	0.15
{a,b,c}	0.55	0.18$\bar{3}$
{a,c,d}	0.60	0.20
{b,c,d}	0.70	0.2$\bar{3}$
{a,b,c,d}	1	0.25

**Table 10:** Belief intervals.

$x_{i}$	$\mathrm{Bel}{(x}_{i})$	$\mathrm{Pl}{(x}_{i})$	$\left[\mathrm{Bel}{(x}_{i}),\mathrm{Pl}{(x}_{i})\right]$
{a}	0.15	0.30	[0.15, 0.30]
{b}	0	0.40	[0, 0.40]
{c}	0	0.70	[0, 0.70]
{d}	0	0.45	[0, 0.45]

**Table 11:** Values of Bel(A) and $\frac{Bel(A)}{\left|A\right|}$.

*A*	*Bel(A)*	$\frac{\mathrm{Bel}(A)}{\left|A\right|}$
{a}	0.10	0.10
{a,b}	0.10	0.05
{a,c}	0.10	0.05
{a,d}	0.10	0.05
{a,e}	0.10	0.05
{b,e}	0.10	0.05
{d,e}	0.20	0.10
{a,b,c}	0.10	0.0$\bar{3}$
{a,b,d}	0.10	0.0$\bar{3}$
{a,b,e}	0.20	0.0$\bar{6}$
{a,c,d}	0.10	0.0$\bar{3}$
{a,c,e}	0.35	0.11$\bar{6}$
{a,d,e}	0.30	0.10
{b,c,d}	0.35	0.11$\bar{6}$
{b,c,e}	0.10	0.0$\bar{3}$
{b,d,e}	0.30	0.10
{c,d,e}	0.20	0.0$\bar{6}$
{a,b,c,d}	0.45	0.1125
{a,b,c,e}	0.45	0.1125
{a,b,d,e}	0.40	0.10
{a,c,d,e}	0.55	0.1375
{b,c,d,e}	0.65	0.15625
{a,b,c,d,e}	1	0.20

**Table 12:** Belief intervals.

$x_{i}$	$\mathrm{Bel}{(x}_{i})$	$\mathrm{Pl}{(x}_{i})$	$\left[\mathrm{Bel}{(x}_{i}),\mathrm{Pl}{(x}_{i})\right]$
{a}	0.10	0.35	[0.10, 0.35]
{b}	0	0.45	[0, 0.45]
{c}	0	0.60	[0, 0.60]
{d}	0	0.55	[0, 0.55]
{d}	0	0.55	[0, 0.55]

**Table 13:** Differences between the two algorithms.

Example	Algorithm	Conflict	Cardinality	Iterations	Maximum of the Entropy
1	Algorithm-1	No	5	3	2.309982
	Algorithm-2		5	2	2.311278
2	Algorithm-1	No	6	4	2.55151
	Algorithm-2		6	2	2.554145
3	Algorithm-1	Yes	4	1	2
	Algorithm-2		4	3	2
4	Algorithm-1	Yes	5	1	2.3219
	Algorithm-2		5	3	2.3219

**Table 14:** Processing time (seconds) for calculating maximum entropy across 1 million randomly generated BPAs for n∈{5,6,10}.

	With Conflict (C)		No Conflict (NC)	
Size	Algorithm-1 (s)	Algorithm-2 (s)	Algorithm-1 (s)	Algorithm-2 (s)
n=5	14.19	16.45	18.39	18.26
n=6	83.32	96.95	85.90	79.54
n=10	23,539.77	30,012.53	24,650.91	23,660.00

## Data Availability

The original contributions presented in this study are included in the article. Further inquiries can be directed to the corresponding author.
